# Effectiveness of Abdominal Deep Breathing Exercises in Managing Blood Pressure Among Hypertensive Patients

**DOI:** 10.7759/cureus.78393

**Published:** 2025-02-02

**Authors:** Akkamahadevi S Bergeri, Shefali S Daruwala

**Affiliations:** 1 Mental Health Nursing, Bharati Vidyapeeth (Deemed to be University) College of Nursing, Sangli, IND

**Keywords:** abdominal deep breathing exercises, assess, blood pressure, effectiveness, hypertensive patients

## Abstract

Introduction: Hypertension remains a leading public health challenge worldwide, requiring innovative and accessible management strategies. Deep breathing exercises have emerged as a promising non-pharmacological intervention, potentially lowering blood pressure through the modulation of the autonomic nervous system. It is suggested that slow and controlled breathing can reduce sympathetic activity and enhance parasympathetic tone, improving cardiovascular function. However, there is limited understanding of the specific physiological mechanisms and sustained effects of deep breathing on blood pressure. This study aims to address this gap by evaluating the efficacy of deep breathing exercises as a complementary approach to hypertension management.

Methodology: The study employed a quantitative, quasi-experimental research design to evaluate the effect of abdominal deep breathing exercises on hypertension. A total of 60 hypertensive patients were selected through a simple random sampling technique, with randomization carried out using a random number table. The participants were divided into two groups: 30 in the experimental group, receiving abdominal deep breathing exercises, and 30 in the control group, receiving standard care. The sample size of 60 was determined based on a power analysis. The inclusion criteria focused on patients with blood pressure levels above 140/90 mmHg, and efforts were made to account for factors like medication use and comorbidities. Data were collected using a standardized tool developed by American Heart Association (AHA) guidelines to ensure accuracy and reliability.

Results: On day one, most patients in both groups had moderate blood pressure. In the experimental group, 13 patients (43%) had moderate systolic blood pressure, compared to 15 patients (50%) in the control group. For diastolic blood pressure, 21 patients (70%) in the experimental group and 20 patients (67%) in the control group had moderate levels. By day seven, the experimental group showed significant reductions in both systolic and diastolic blood pressure compared to the control group. The mean systolic blood pressure in the experimental group dropped to 141.66 mmHg (standard deviation (SD) 15.99), and the mean diastolic blood pressure decreased to 92.66 mmHg (SD 10.81). These results indicate that abdominal deep breathing exercises effectively lower blood pressure.

Conclusion: The study concludes that abdominal deep breathing exercises are an effective non-drug method to lower blood pressure in hypertensive patients. The significant reduction in both systolic and diastolic blood pressure highlights the potential benefits of including deep breathing exercises in regular hypertension management. However, further studies with larger sample sizes and longer durations are needed to confirm these findings and ensure the long-term effectiveness of this approach.

## Introduction

Hypertension is often undiagnosed and untreated until complications, such as cardiovascular and cerebrovascular diseases, develop. It is defined by elevated systolic (≥140 mmHg) and diastolic (≥90 mmHg) blood pressure (BP), influenced by factors such as blood volume, velocity, and cardiac output. Over the past four decades, the prevalence of hypertension has risen, with urban populations being more affected than rural ones. Health is a multifaceted concept encompassing physical, social, psychological, and spiritual well-being. It is shaped by determinants such as housing, education, income, employment, and access to healthcare services. These factors collectively affect an individual’s ability to manage hypertension and overall health, highlighting the need for targeted interventions and equitable resource distribution to address these disparities and improve health outcomes.

Elavarasi's study concluded that abdominal breathing exercises are an effective, non-pharmacological intervention for regulating BP in hypertensive patients. The results demonstrated a significant reduction in BP levels among participants who practiced abdominal breathing exercises compared to those who did not. This suggests that incorporating such exercises into routine care can be a simple, cost-effective strategy to improve hypertension management. The study emphasized the importance of lifestyle modifications and highlighted the potential of abdominal breathing exercises to complement existing treatment modalities, enhancing overall cardiovascular health and quality of life for patients with hypertension [1].

The study by Arvind Singh et al. concluded that abdominal breathing exercises are highly effective in reducing mean BP among hypertensive patients. The pre- and post-assessment results demonstrated a significant decrease in BP readings, with a mean difference of 5.61 mmHg and a t-value of 19.39, indicating statistical significance. These findings suggest that incorporating abdominal breathing exercises into the management plan for hypertensive patients can be a beneficial, non-pharmacological intervention to help control BP levels [2].

The study by Amandeep et al. concluded that abdominal breathing exercises are an effective complementary therapy for reducing BP among hypertensive patients. Conducted with 60 primary hypertensive subjects, the research demonstrated a significant reduction in BP in the experimental group (p = 0.00). Additionally, the study found a significant association between BP levels and the age of hypertensive patients. These findings suggest that incorporating abdominal breathing exercises into hypertension management can be a beneficial, non-pharmacological approach to controlling BP [3].

The study by Vasuki and Sweety concluded that regular practice of deep breathing exercises is effective in reducing both systolic and diastolic BP among pre-hypertensive and hypertensive patients. The research demonstrated a significant decrease in BP readings in the experimental group compared to the control group, with beneficial effects becoming evident as early as four weeks into the intervention. This suggests that incorporating deep breathing exercises can enhance parasympathetic activity and reduce sympathetic excitability, offering a non-pharmacological approach to managing hypertension [4].

Abdominal deep breathing exercises can be an effective, non-pharmacological approach to managing hypertension. These exercises help reduce chronic stress and tension-key contributors to elevated BP-by promoting relaxation and slowing the pulse. Additionally, deep breathing may support more efficient processing of salt in the body, which is crucial for BP regulation. Regular practice of deep breathing improves blood and oxygen flow to the brain, enhancing its function. Multiple studies consistently show that deep and abdominal breathing exercises can reduce BP, improve cardiovascular health, and promote overall well-being. Given these benefits, integrating deep breathing exercises into routine care for hypertensive patients offers a valuable complementary strategy alongside pharmacological treatments, supporting long-term health management.

## Materials and methods

The study employed a quantitative research approach with a quasi-experimental design to evaluate the effectiveness of abdominal deep breathing exercises on BP among hypertensive patients. Ethical approval was obtained from the institutional ethics committee, ensuring adherence to ethical guidelines throughout the study. The research targeted hypertensive patients with BP levels exceeding 140/90 mmHg, excluding those with respiratory or gastrointestinal disorders. A total of 60 participants, including male and female participants, were selected using a simple random sampling technique and divided into 30 in the experimental group and 30 in the control group. To ensure accuracy and reliability, a data collection tool was developed based on standardized guidelines from the American Heart Association (AHA), with BP measured using a calibrated sphygmomanometer and stethoscope. The tool's reliability was verified through the test-retest method, yielding a reliability coefficient (r = 0.9618) calculated using Karl Pearson's formula, indicating high reliability. Written informed consent was obtained from all participants before their inclusion.

The study was conducted in three phases: The pre-intervention phase involved collecting demographic data to establish baseline characteristics in both groups. During the intervention phase, participants in the experimental group had their BP monitored before being instructed to perform abdominal deep breathing exercises for 30 minutes twice daily (morning and evening) for seven consecutive days, while the control group continued to receive standard hospital care without additional intervention. The post-intervention phase involved reassessing BP in both groups on the eighth day using the same calibrated tools. The abdominal deep breathing exercise procedure was conducted in a designated area with beds arranged for participants, who were prepared and given clear instructions to sit upright with one hand on the abdomen and the other on the chest; inhale deeply through the nose for a count of four, ensuring the abdomen rises higher than the chest; hold the breath for a count of seven; and exhale slowly through the nose for a count of eight. The exercise was demonstrated by the investigator, and participants practiced under supervision for 30 minutes twice daily over seven days, with advice to continue the practice after the intervention to maintain normal BP levels.

The institutional ethics committee of Bharati Vidyapeeth (Deemed to be University), College of Nursing, Sangli, approved the study (IECBVDUCON, Sangli Maharashtra Reg. No. EC/NEW/INST/2024/MH/0414). Informed consent was obtained from all participants, who were thoroughly briefed about the study's objectives and clarified that participants were informed of their right to withdraw at any time without penalty through the informed consent form, which was reviewed and signed before participation. Data analysis involved both descriptive and inferential statistics to summarize baseline characteristics and assess the effectiveness of the intervention. Mean and standard deviation (SD) values were calculated to evaluate pre- and post-intervention BP levels.

The study was organized into four sections for data analysis and interpretation. Section 1 focused on the frequency and percentage distribution of demographic variables, providing a baseline understanding of the participants' characteristics. Section 2 assessed the level of BP among hypertensive patients before the intervention, offering insights into the pre-intervention status of the participants. Following the intervention, Section 3 looked at the BP levels of hypertensive patients, emphasizing any changes that were ascribed to the tactics used. Finally, Section 4 compared the post-test scores of the experimental and control groups, revealing significant differences and emphasizing the effectiveness of abdominal deep breathing exercises in managing hypertension.

## Results

In both the experimental and control groups, the majority of participants were aged 51-60 years, with 10 participants (33%) in the experimental group and nine participants (30%) in the control group. Gender distribution revealed that most participants in the experimental group were men, with 20 (67%), while the control group predominantly consisted of women, with 18 (60%). Regarding the duration of hypertension, the majority of participants, 17 (56%) in the experimental group and 18 (60%) in the control group, had been living with hypertension for one to five years. Additionally, all hypertensive patients, totaling 30 (100%) in both the experimental and control groups, were on medication to manage their condition. The frequency and percentage distribution of demographical variables are shown in Table 1.

**Table 1:** Frequency and percentage distribution of demographical variables f: frequency; %: percentage

Sr. No.	Demographic variables		Experimental group		Control group	
			f	%	f	%
1	Age (in years)	21-30	3	10%	2	7%
		31-40	3	10%	6	20%
		41-50	7	23%	5	17%
		51-60	10	33%	9	30%
		61-70	5	17%	5	17%
		71 & above	2	7%	3	10%
2	Gender	Male	20	67%	12	40%
		Female	10	33%	18	60%
3	Duration of hypertension	Below 1 year	0	0%	0	0%
		1-5 years	17	56%	18	60%
		6-10 years	11	37%	9	30%
		11-15 years	2	7%	3	10%
		16-20 years	0	0%	0	0%
		21 & above years	0	0%	0	0%
4	Medication	Taking	30	100%	30	100%
		Not taking	0	0%	0	0%

Significant results were obtained by observing the systolic and diastolic BP ranges of hypertensive patients in the experimental and control groups prior to the administration of abdominal deep breathing exercises. Systolic BP on day one was within a moderate range for the majority of patients, with 13 (43%) in the experimental group and 15 (50%) in the control group. Similarly, most patients, 21 (70%) in the experimental group and 20 (67%) in the control group, demonstrated a moderate range of diastolic BP. These baseline measurements provided a foundation for assessing the effectiveness of the intervention. The level of BP among hypertensive patients before intervention is shown in Table 2.

**Table 2:** Assessment of the level of blood pressure among hypertensive patients before intervention f: frequency; %: percentage

Group type	Systolic blood pressure								Diastolic blood pressure							
	<140 normal stage		140-159 (stage 1)		160-179 (stage 2)		>180 (hypertensive crisis)		<90 normal stage		90-99 (stage 1)		100-119 (stage 2)		>120 (hypertensive crisis)	
	f	%	f	%	f	%	f	%	f	%	f	%	f	%	f	%
Experimental group	0	0	12	40	13	43	5	17	0	0	2	7	21	70	7	23
Control group	0	0	13	43	15	50	2	7	0	0	5	17	20	67	5	17

By day seven, there were notable differences between the systolic and diastolic BP ranges of hypertensive patients in the experimental and control groups following the administration of abdominal deep breathing exercises. In the experimental group, most patients, 13 (43%), experienced a mild range of systolic BP, while 12 (40%) achieved a normal range. In contrast, the majority of patients in the control group, 20 (67%), remained in the mild range of systolic BP. Regarding diastolic BP, most patients in the experimental group, 21 (70%), experienced a mild range, with three (10%) achieving a normal range. Meanwhile, the majority of patients in the control group, 22 (73%), continued to exhibit a moderate range of diastolic BP. These results underscore the impact of abdominal deep breathing exercises on improving BP control in the experimental group, providing a more detailed and reliable presentation of the changes observed. The level of BP among hypertensive patients after intervention is shown in Table 3.

**Table 3:** Assessment of the level of blood pressure among hypertensive patients after intervention f: frequency; %: percentage

Group type	Systolic blood pressure								Diastolic blood pressure							
	<140 normal stage		140-159 (stage 1)		160-179 (stage 2)		>180 (hypertensive crisis)		<90 normal stage		90-99 (stage 1)		100-119 (stage 2)		>120 (hypertensive crisis)	
	f	%	f	%	f	%	F	%	f	%	f	%	f	%	f	%
Experimental group	12	40	13	43	3	10	2	7	3	10	21	70	5	17	1	3
Control group	0	0	20	67	8	27	2	7	0	0	6	20	22	73	2	7

The experimental and control groups showed a significant difference in their systolic and diastolic BP on day seven, after the administration of abdominal deep breathing exercises. The experimental group's mean systolic BP was 141.66 with an SD of 15.9921, whereas the control group's was higher at 154 with an SD of 14.3077. The calculated t-value was 3.2331, with a p-value of 0.002, indicating a statistically significant difference (p < 0.05). Similarly, for diastolic BP, the experimental group recorded a mean of 92.66 with an SD of 10.8065, whereas the control group had a mean of 101.66 with an SD of 14.4038. The t-value was 2.7375, and the p-value was 0.0082, also showing a statistically significant difference (p < 0.05). These findings highlight the effectiveness of abdominal deep breathing exercises in reducing both systolic and diastolic BP among hypertensive patients in the experimental group. The comparison of post-test scores in the experimental and control groups is shown in Table 4.

**Table 4:** Comparison of post-test scores in the experimental and control groups SD: standard deviation

Blood pressure	Group	Mean	SD	t-value	p-value	Significance
Systolic	Experimental	141.66	15.9921	3.2331	0.002	Significant
	Control	154.33	14.3077			
Diastolic	Experimental	92.66	10.8065	2.7375	0.0082	Significant
	Control	101.66	14.4038			

## Discussion

The present study aimed to evaluate the effectiveness of abdominal deep breathing exercises on BP among hypertensive patients. The findings demonstrated a statistically significant reduction in both systolic and diastolic BP levels in participants who regularly practiced the intervention, compared to those in the control group. This reduction in BP is attributed to the physiological effects of abdominal deep breathing, which enhances parasympathetic nervous system activation and reduces sympathetic activity. These changes promote vasodilation and improve heart rate (HR) variability. Furthermore, the deliberate breathing pattern helps to mitigate stress-induced catecholamine release, a key factor contributing to elevated BP in hypertensive individuals. The results highlight the potential of abdominal deep breathing exercises as a cost-effective, non-invasive, and easily implementable approach for BP management. Incorporating this intervention into routine clinical practice or community health programs could be particularly beneficial for individuals seeking alternative or complementary methods to pharmacological treatments.

The study by Wang et al. found that slow abdominal breathing, when combined with electromyographic (EMG) biofeedback, effectively manages pre-hypertension. Participants in this intervention experienced significant reductions in both systolic and diastolic BP, as well as improved HR variability. The authors propose that this combination may reduce sympathetic activity and enhance vagal activity, thus aiding better BP regulation. These findings are consistent with other research suggesting that non-pharmacological interventions, such as deep breathing exercises and biofeedback, can effectively manage hypertension. Integrating such approaches into routine care presents a promising, cost-effective strategy for controlling BP and improving cardiovascular health [5].

The study by Salian and Gireesh concluded that abdominal breathing exercises effectively reduce BP, HR, and respiration rate (RR) among hypertensive patients. The experimental group, which practiced these exercises, exhibited significant improvements in these biological parameters compared to the control group. This suggests that incorporating abdominal breathing exercises into hypertension management can be a beneficial, non-pharmacological approach to controlling BP and enhancing overall cardiovascular health [6].

A similar study by Mohammad et al. concluded that deep breathing exercises significantly lower BP among hypertensive patients. The research demonstrated a substantial reduction in hypertension levels, with a mean decrease of 13.34. points, indicating the effectiveness of this non-pharmacological intervention. Additionally, the study found significant associations between hypertension levels and factors such as age, gender, education, occupation, duration since diagnosis, and family history of diabetes mellitus. These findings suggest that incorporating deep breathing exercises into hypertension management can be a beneficial strategy to control BP and improve cardiovascular health [7].

Ola's study focused on the implementation of breathing exercises at home for hypertension patients aged 36-45 years. The research demonstrated that consistent practice of deep breathing exercises led to significant reductions in both systolic and diastolic BP. Participants showed a mean reduction of 30.46 mmHg in systolic and 13.65 mmHg in diastolic pressure. This non-pharmacological approach offers an accessible and effective method for managing hypertension, especially when integrated into daily routines. The findings support the growing body of evidence suggesting the benefits of breathing exercises in controlling BP and enhancing cardiovascular health [8].

The study by Verma and Verma concluded that deep breathing exercises significantly reduce BP among hypertensive individuals. Participants who engaged in these exercises experienced notable decreases in both systolic and diastolic BP, highlighting the effectiveness of this non-pharmacological intervention. These findings align with other research indicating that breathing exercises can positively impact BP regulation [9].

The study by Hoesny et al. concluded that deep breathing therapy significantly reduces BP among hypertensive patients. Participants who engaged in deep breathing exercises experienced notable decreases in both systolic and diastolic BP, highlighting the effectiveness of this non-pharmacological intervention. These findings align with other research indicating that breathing exercises can positively impact BP regulation. Incorporating deep breathing exercises into daily routines offers a simple, cost-effective strategy for managing hypertension and improving cardiovascular health [10].

A limitation of this study is the relatively small sample size, which may limit the generalizability of the findings. Additionally, the study was conducted at a single healthcare facility, which may not account for the diversity of populations and settings. The study also relied on self-reported adherence to the breathing exercises, which could introduce biases such as recall or social desirability bias. These biases may affect the accuracy and reliability of the results. Future studies should include larger and more diverse samples to provide a more comprehensive evaluation. We will discuss these limitations in more detail and explore how they could impact the clinical applicability and scalability of the intervention. A more thorough examination of potential biases and their influence on the outcomes will also be included in the discussion.

## Conclusions

The study concludes that, in India, hypertension is becoming increasingly common and poses a significant public health concern. It is a dangerous condition that can cause lasting damage to the heart and significantly increase the risk of death and illness. This study demonstrates that abdominal deep breathing exercises are an effective, low-cost intervention for managing hypertension. The results showed a clear reduction in both systolic and diastolic BP. Specifically, systolic BP decreased by an average of 13 mmHg (p < 0.05), and diastolic BP reduced by 7 mmHg (p < 0.05), the potential of these exercises as an effective tool in hypertension management. However, the study's small sample size and single healthcare facility setting limit the generalizability of these findings to the broader population. The observed reductions in BP may be effective in the short term, but further research is needed to assess the sustainability of these effects over time. These findings align with previous research, which also supports the positive impact of breathing exercises on BP. While incorporating abdominal deep breathing into daily routines may provide significant benefits for people with hypertension, the feasibility of such an intervention in real-world settings needs further exploration. For instance, in India, barriers like accessibility, cultural acceptance, and the need for proper training may influence the effectiveness of this intervention. The intervention's low-cost nature is appealing, but additional resources for training and support may be necessary to ensure its widespread adoption. The potential for integrating abdominal deep breathing into clinical practices or public health programs could offer a simple yet powerful addition to current hypertension treatment plans. However, long-term adherence and reinforcement strategies will be essential to maintaining its effectiveness.
